# Whole-genome sequencing of *Shigella* for surveillance purposes shows (inter)national relatedness and multidrug resistance in isolates from men who have sex with men

**DOI:** 10.1099/mgen.0.000978

**Published:** 2023-04-06

**Authors:** Maaike van den Beld, Roan Pijnacker, Alje van Dam, Lian Bovée, David Kwa, Ineke Linde, Roxanne Wolthuis, Daan Notermans, Thijs Bosch, Eelco Franz

**Affiliations:** ^1^​ Centre for Infectious Disease Control, National Institute for Public Health and the Environment, Bilthoven, Netherlands; ^2^​ Department of Infectious Diseases, GGD Amsterdam, Amsterdam, Netherlands; ^3^​ Department of Medical Microbiology and Infection Prevention, Amsterdam UMC, Amsterdam, Netherlands; ^4^​ Department of Medial Microbiology, OLVG Laboratories, Amsterdam, Netherlands

**Keywords:** *Shigella*, pathogen surveillance, surveillance, whole-genome sequencing, antimicrobial resistance, MSM

## Abstract

In the Netherlands, more than half of domestic shigellosis cases are among men who have sex with men (MSM), particularly in the Amsterdam region. However, there is limited insight into which *Shigella* strains circulate in the Netherlands. Our objective was to assess the added value of whole-genome sequencing (WGS)-based surveillance for *Shigella *. To this end, we determined the relatedness among *Shigella* spp. isolates from patients in the Amsterdam region, as well as in an international context, including antimicrobial resistance markers, using WGS. The following criteria were used: it should provide insight into (1) clustering of shigellosis cases and the affected population, (2) the extent of admixture of MSM-associated isolates with those from the broader population and (3) the presence of antimicrobial resistance. It should then lead to more opportunities for targeted control measures. For this study, *Shigella* isolates from three laboratories in the Amsterdam region obtained between February 2019 and October 2021 were subjected to Illumina WGS at the National Institute for Public Health and the Environment (RIVM). Raw data were quality-checked and assembled, the *Shigella* serotype was determined with ShigaTyper, and antimicrobial resistance markers were detected using ResFinder and PointFinder. For *Shigella sonnei *, subclades were determined using Mykrobe. Relatedness of isolates, including 21 international reference genomes, was assessed with core genome multilocus sequence typing. In total, 109 isolates were included, of which 27 were from females (25 %) and 66 were from males (61 %), with which the majority (*n*=48, 73 %) being from MSM. No information on sex was available for the remaining 16 cases. The WGS data for all isolates, comprising 55 *S *. *sonnei *, 52 *Shigella flexneri *, 1 *Shigella boydii* and 1 *Shigella dysenteriae *, met the quality criteria. In total, 14 clusters containing 51 isolates (49 %) were identified, with a median cluster size of 2.5 cases (range: 2–15). Nine out of 14 clusters were MSM-associated, and 8 clusters (57 %) were travel-related. Six of the MSM clusters were related to international reference genomes. The prevalence of antimicrobial resistance markers was higher among isolates from MSM than non-MSM patients, particularly for ciprofloxacin (89 vs 33 %) and azithromycin (58 vs 17 %). In conclusion, about half of *Shigella* spp. patients were part of a cluster, of which a substantial part were related to international reference genomes, particularly among MSM, and a high prevalence of antimicrobial resistance markers was found. These findings indicate widespread international circulation of *Shigella* spp., particularly among MSM, with multidrug resistance that hampers treatment of patients. Moreover, the results of this study led to the implementation of a national WGS-based laboratory surveillance programme for *Shigella* spp. that started in April 2022.

## Data Summary

All raw sequences were submitted to the European Nucleotide Archive under study number PRJEB56557. The authors confirm that all supporting data, code and protocols have been provided within the article or through supplementary data files.

**Impact Statement**

*
Shigella* is on the priority list of the World Health Organization regarding antimicrobial resistance, notably against ciprofloxacin and third-generation cephalosporins. The literature stated that multidrug-resistant *Shigella* isolates are frequently encountered in multiple countries, especially among men who have sex with men (MSM). In the Netherlands, pathogen surveillance of shigellosis is not employed, and the characteristics of circulating isolates and their place in the global context is unknown. Epidemiological investigations alone, such as contact and source tracing, are often difficult, especially amongst MSM. To be able to take control measures to prevent further spread of mainly (multidrug-) resistant isolates, it is important to understand which groups are mostly affected and identify circulating clades. This sentinel surveillance study showed that multiple multidrug-resistant *Shigella* strains circulate in the Netherlands, particularly among MSM, of which a substantial part were related to international MSM-associated clades. This study highlighted the need for national genomic pathogen surveillance for *Shigella* and, as a result, a national surveillance programme was implemented. This aids the Netherlands and other countries in their control of shigellosis by enabling identification of antimicrobial-resistant emerging clades of interest at an early stage in a global setting.

## Introduction

Shigellosis, also called bacillary dysentery, is caused by an intestinal infection with the bacterium *Shigella *. The genus *Shigella* constitutes the four species *Shigella dysenteriae, Shigella flexneri, Shigella boydii* and *Shigella sonnei *, which can all cause human illness. Shigellosis often manifests as watery and bloody diarrhoea, tenesmus and fever [1]. *S. flexneri* and *S. sonnei* are the species most encountered in the Netherlands [2]. *Shigella* is a highly infectious pathogen, with humans as the only natural reservoir [3]. Transmission occurs through the faecal–oral route, via direct human-to-human (faecal–oral) transmission, or indirectly via contaminated food or water [4]. Additionally, transmission through sexual contact, specifically amongst men who have sex with men (MSM), is frequently reported [4, 5].

In the Netherlands, physicians and laboratories are required by law to notify all shigellosis cases in which an isolate has been cultured to the municipal health authorities (MHS). For each case, contact and source tracing investigation is performed by the MHS and the resulting pseudonymized data are registered as part of national surveillance at the National Institute for Public Health and the Environment (RIVM). In the period 1988 to 2015, an annual average of 391 cases (range: 244–598) of shigellosis were reported in the Netherlands, of which most (73 %) were acquired abroad. Between 2006 and 2010, an increasing number of infections were domestic, while the number of travel-related infections did not change [4]. After 2010, the number of domestic shigellosis cases plateaued, showing the highest incidence among male patients aged 30–65 years [4]. The increase in domestic infections could be attributed to sexual transmission between MSM, who comprised 63 % of adult male shigellosis patients since 2010 [4].

To our knowledge, the UK is the only country in Europe that routinely applies whole-genome sequencing (WGS) for national *Shigella* pathogen surveillance [6, 7], and it has reported multiple clusters and/or outbreaks of *Shigella* that are MSM-related [5, 8, 9]. From countries outside Europe or from studies from other European countries there is more insight into circulating strains. In Switzerland, three cases of sexually transmitted shigellosis were described, all three related to UK clusters [10]. In 2021, the circulation of multidrug-resistant *Shigella* was reported by Belgium and Spain, which were related to MSM clusters in other countries inside and outside Europe [11, 12]. An increasing number of studies report that *Shigella* isolates from MSM-related clusters are more often resistant to multiple antimicrobials compared with other subpopulations [9, 13, 14]. *Shigella* that are resistant to third-generation cephalosporins and ciprofloxacin are considered to be a global public health concern by the World Health Organization (WHO) [15].

In the Netherlands, there is no insight into the presence of these internationally circulating MSM-associated strains, because of the absence of a (WGS-based) pathogen surveillance system for *Shigella *. However, data from a multicentre study on *Shigella* and enteroinvasive *Escherichia coli* indicated that the majority of isolates are part of clusters and that MSM-associated isolates are more resistant to antimicrobials. Source and contact tracing was found to be challenging without underlying high-resolution typing data, especially amongst MSM, partially due to the potential for anonymous sexual contact [13]. The results of this former study indicated the spread of MSM-associated strains to men that do not have sex with men or women [13]. The spread of MSM infections to the broader population was described before for hepatitis A in the Netherlands [16].

In order to determine the added value of a routinely applied national genomic surveillance of *Shigella *, a WGS-based pilot sentinel surveillance study was conducted from 2019 to 2021 in the Amsterdam region. Its added value was assessed using internally predefined criteria for implementing such a national genomic surveillance programme. First, the pilot study should provide increased insight into clustering of shigellosis cases in the Netherlands, but also with internationally described clusters, as well as the affected population. Second, it should enable us to determine the extent to which spreading of isolates occurred from MSM to the broader population. Third, the presence of antimicrobial resistance among the isolates concerning public health according to the WHO should be identified in the pilot study. Fourth and last, genomic pathogen surveillance should potentially lead to more targeted control measures by the MHS during outbreaks. The objectives of this pilot study were therefore to identify whether these criteria were met, with the aim of informing the decision to implement a national genomic surveillance programme for *Shigella* based on WGS that would support the current shigellosis surveillance system.

## Methods

### Study design and population

This pilot sentinel surveillance study was conducted within the Amsterdam region because an earlier study showed that 79 % (61/77) of shigellosis amongst MSM in the Netherlands was reported from this region [2]. The study was initially set to last for 2 years, from March 2019 to March 2021, but was extended to October 2021 due to lower case numbers as a result of the coronavirus disease 2019 (COVID-19) pandemic. Three laboratories in the Amsterdam region participated in the study, including OLVG laboratories, Public Health Laboratory GGD Amsterdam and Amsterdam University Medical Centre. They sent culture-confirmed *Shigella* isolates to the RIVM for WGS. The MHS Amsterdam performed contact and source tracing and collected information on e.g. sex with other men (in the case of male patients) and travel history, and reported this information to the RIVM, as part of the standard protocol. *Shigella* isolates sent in by participating laboratories were matched manually to notifications from the surveillance system at RIVM based on the sex, year of birth and four-digit postal code of the patient. If multiple isolates from the same patient were sent in, only the first one was used.

### Isolates and typing

Illumina sequencing and assembly were performed for all cultured *Shigella* isolates that were sent to the RIVM for WGS, as described before using the in-house pipeline Juno-assembly v2.0.6. based on SPAdes version 3.15.3 [17]. Raw reads with a Phred score ≥30 and *de novo* assemblies with a total length of 4.21–5.03 Mbp, an N50 >30 000, a GC percentage between 50.3 and 51.0 %, contig number <762, genome completeness >96 % and contamination level <4 % were considered to be of good quality and used in further analyses. All tools used in the analyses were with default parameters unless stated otherwise. *In silico Shigella* serotyping based on ShigaTyper 1.6.0 [18] and *in silico E. coli* O and H serotyping based on SeroTypeFinder commit d23ee1b [19] were performed using an in-house-developed pipeline called Juno-typing v0.5.0 [20]. Detection of AMR markers was conducted using an in-house-developed pipeline, Juno-AMR v0.4 [21], based on ResFinder and PointFinder version 4.1.3 [22, 23]. Isolates were considered multidrug-resistant if genetic markers predicted resistance against ≥3 classes of the antimicrobials quinolones, extended beta-lactamases, macrolides, sulfonamides and trimethoprim. Core genome multilocus sequence typing (cgMLST) was performed using the *de novo* assemblies in Ridom SeqSphere 8.3.1 with the *Escherichia */* Shigella* v1 scheme of Enterobase, consisting of 2513 alleles. Distances were calculated using Hamming distances, ignoring pairwise missing alleles. Using these distances, clusters were determined using single-linkage hierarchical agglomerative clustering. Isolates were considered to comprise a cluster if the distance was ≤5 alleles. To determine if cases were part of international clusters, reference sequences from described MSM-associated clusters from Australia [14], Belgium [11], Spain [12] and the UK [5, 24] were downloaded and added to the cgMLST analysis (Table S1, available with the online version of this article). To describe relatedness with international clusters, a cut-off of a maximum of 20 allelic differences (AD) was used. Additionally, for *S. sonnei *, isolates were genotyped according to the genotyping framework described by Hawkey *et al*. using Mykrobe v0.9.0+ [25, 26]

### Descriptive analysis

Cases were described by age (median and range), sex, sexual activity (for males only; MSM or non-MSM), travel history and infecting *Shigella* species. The same was described for clusters identified based on cgMLST, of which the description was further stratified by pre-COVID-19 and during the COVID-19 pandemic. A cgMLST cluster was considered to be MSM-associated if ≥50 % of included cases reported MSM contact and a cluster was considered travel-related if at least ≥50 % of isolates were from patients that acquired the infection abroad. MSM-associated clusters that also contained female cases without travel history were considered to be an indication for spreading of MSM-related isolates to the broader population. Furthermore, we assessed the relatedness of these same clusters with international reference genomes of MSM-associated clusters, as well as the prevalence of genetic antimicrobial resistance markers in different subpopulations (e.g. by cluster, MSM/non-MSM). A neighbour-joining dendogram was used to visualize isolate relatedness and cluster characteristics using iTol, stratified by the two most common species *S. sonnei* and *S. flexneri* [27]. Analyses were performed in STATA version 17.0 (StataCorp, TX, USA). When testing for statistical significant differences between groups of patients, N-1 chi-squared tests were used to compare proportions.

## Results

### Included cases and epidemiology

The RIVM received 117 isolates from 115 patients from March 2019 until October 2021. This was 13.6 % of the total 843 shigellosis cases that were reported in that time period in the Netherlands. For each unique patient, one isolate was subjected to WGS. One of the isolates was not identified as *Shigella* and the sequences of five isolates did not meet the predetermined quality criteria, resulting in the inclusion of 109 *Shigella* isolates of good quality (Table 1). Of those, 55 (50 %) were *S. sonnei *, 52 (48 %) *S *. *flexneri *, 1 *S. boydii* and 1 *S. dysenteriae *. For 93 (85 %) of these isolates, a corresponding case was found in the surveillance system.

**Table 1.** Species of 109 included cases, of which 42 had a travel history and 48 identified as men who have sex with men (MSM), February 2019–October 2021

	Total (*n*=109)		Travel history (*n*=42)		MSM identified (*n*=48)	
	n	%	n	%	n	%
Species						
* S. sonnei *	55	50	24	57	19	40
* S. flexneri *	52	48	16	38	29	60
* S. boydii *	1	1	1	2	0	0
* S. dysenteriae *	1	1	1	2	0	0

In the period before the COVID-19 pandemic (February 2019 to February 2020), isolates were obtained less frequently from MSM (*n*=32, 41 %) than during the pandemic (*n*=16, 53 %; March 2020 until October 2021), although this was not statistically significantly different (p=0.262) (see Table 2). Before the COVID-19 pandemic, isolates were more often travel-related (46 %) than during the pandemic (20 %, p=0.013).

**Table 2.** Description of cases by sex, sexual activity (i.e. MSM/non-MSM) and country of infection, stratified by isolates that cluster and isolates that do not cluster with at least one other isolate, for the period before and the period during the COVID-19 pandemic

	Cases		Clustered isolates*		Singletons		Pre-COVID-19†		During COVID-19‡	
	*n*=109		*n*=51		*n*=58		*n*=79		*n*=30	
	n	%	n	%	n	%	n	%	n	%
Sex										
Male	66	61	39	76	27	47	46	58	20	67
Female	27	25	6	12	21	36	21	27	6	20
Unknown	16	15	6	12	10	17	12	15	4	13
MSM (among males)										
Yes	48	44	29	57	19	33	32	41	16	53
No	17	16	9	18	8	14	14	18	3	10
Unknown	1	1	1	2	0	0	0	0	1	3
Country of infection										
Netherlands	48	44	30	59	17	29	29	37	19	63
Elsewhere	42	39	15	29	29	50	36	46	6	20
Unknown	19	17	6	12	12	21	14	18	5	17

*Clusters with at least one other isolate based on cgMLST.

†February 2019–February 2020.

‡March 2020–October 2021.

### Clustering using cgMLST

In total, 14 clusters were identified based on cgMLST with a cluster cut-off of ≤5 alleles, including approximately half of the total number of cases (*n*=51, 49 %). Clusters had a median size of 2.5 cases (range: 2–15 cases). Isolates that clustered with at least one other isolate were more frequently from MSM than from non-MSM (*P*=0.012), and from domestic cases than travel-related cases (p=0.002,Table 2).

**Fig. 1.**
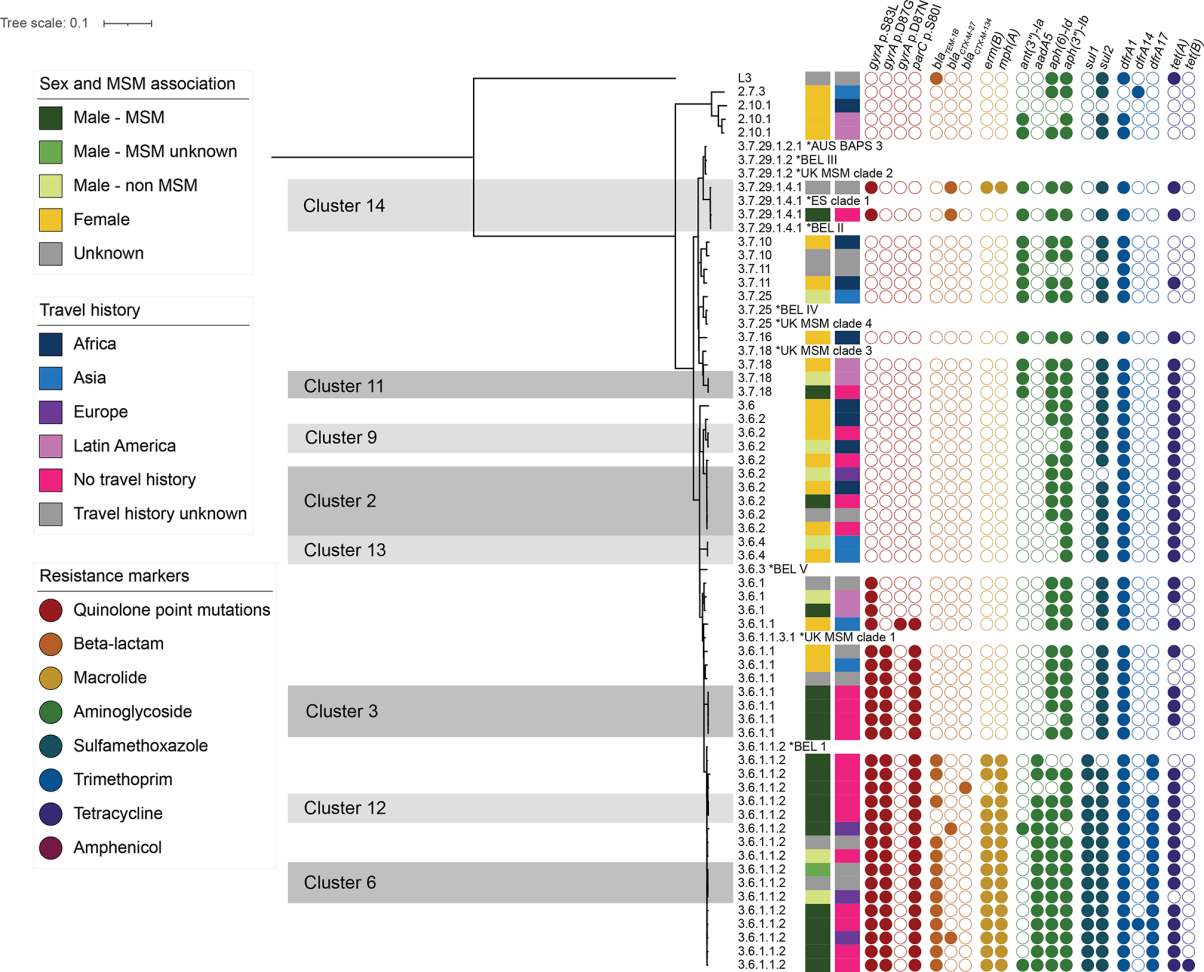
Neighbour-joining tree for *Shigella sonnei* isolates, including international reference genomes (*), based on cgMLST. Grey areas represent clusters within the cluster cut-off of ≤5 alleles.

**Fig. 2.**
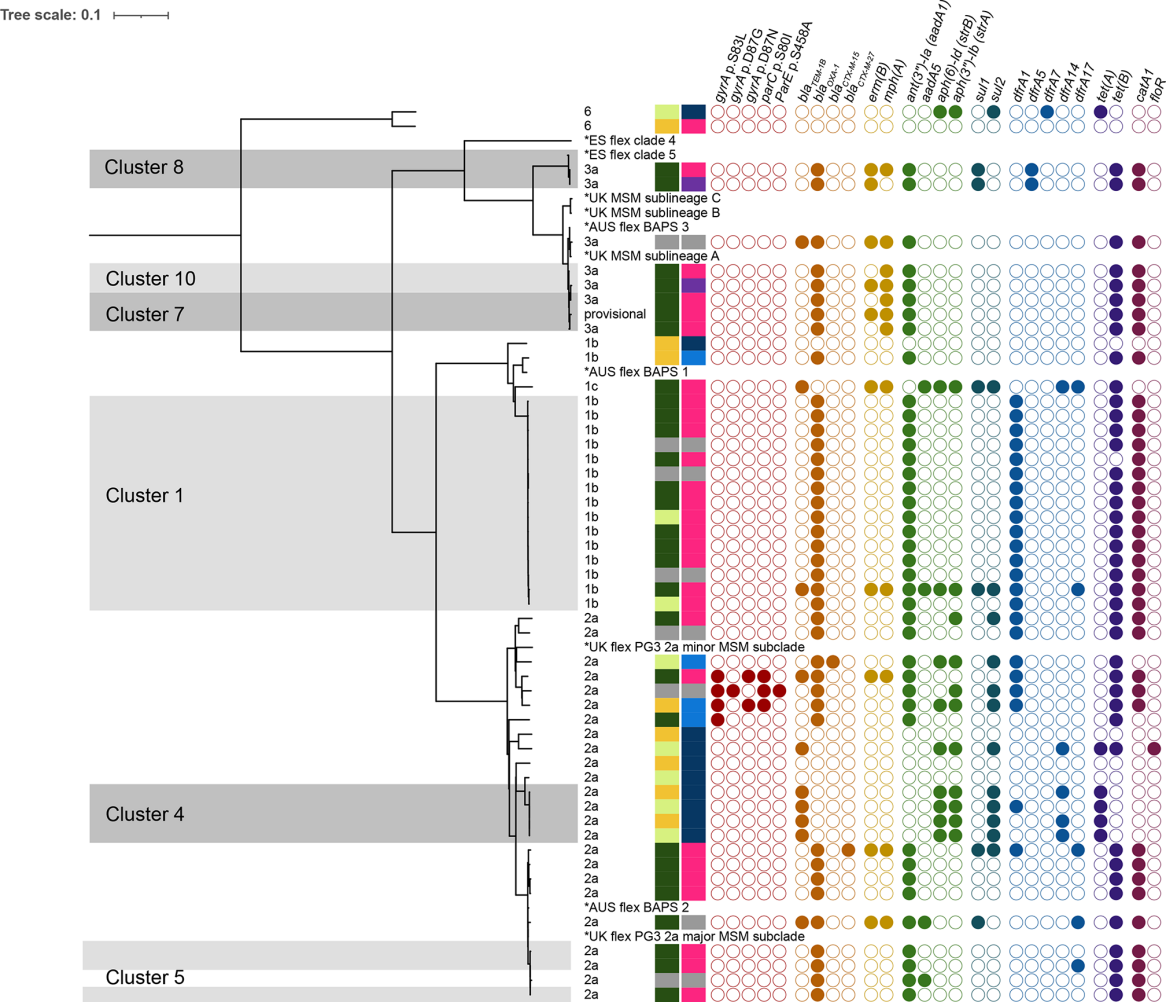
Neighbour-joining tree for *Shigella flexneri* isolates, including international reference genomes, based on cgMLST. Grey areas represent clusters within the cluster cut-off of ≤5 alleles.

Eight out of 14 clusters were *S. sonnei* and six were *S. flexneri* with serotypes 2 a, 3 a and 1b (Table 3 and Figs 1 and 2). For nine clusters, at least 50 % of cases reported MSM contact, of which seven consisted exclusively of isolates from MSM. Of those nine MSM-related clusters, six (67 %) were within 20 AD from international reference isolates (Table 3) and two were related to MSM reference isolates within the cluster cut-off of ≤5 alleles. Eight clusters contained at least 50 % travel-related isolates, of which two clusters consisted exclusively of isolates from cases that travelled. One of the latter clusters consisted of *S. flexneri* serotype 2 a (cluster 4) and was linked to the Republic of Ghana. Cluster 13 was a travel-related *S. sonnei* cluster with subclade 3.6.4 linked to Sri Lanka. There was one cluster (cluster 2) where admixing of MSM-associated isolates with the broader population was observed, which included two males (one MSM) and two females.

**Table 3.** Characteristics of clusters identified with cgMLST using a cut-off of ≤5 alleles, including their relatedness to international MSM-associated reference genomes

ID	#isolates	Species	Serotype SF/	Male/female	MSM*	Travel history	Related to international
			genotype SS		yes/no		MSM references/subclades
1	15†	* S. flexneri *	1b	12/0†	10-Feb	None	No
2	5‡	* S. sonnei *	3.6.2	2/2‡	01-Jan	1 x Morocco,	No
						1 x Greece	
3	4	* S. sonnei *	3.6.1.1	4/0	4/0	None	No
4	4	* S. flexneri *	2a	02-Feb	0/2	4 x The Ghana	No
5	3	* S. flexneri *	2a	3/0	3/0	None	UK_Major MSMclade (10 AD); Australia_flexneri_BAPS2 (15 AD)
6	3‡	* S. sonnei *	3.6.1.1.2	2/0‡	0/1‡	1 x Spain	BEL_I (8 AD)
7	3	* S. flexneri *	3a	3/0	3/0	None	UK_MSM sublineageA (13 AD); Australia_flexneri_BAPS3 (18 AD)
8	2	* S. flexneri *	3a	2/0	2/0	1 x Spain	Spain_flexneri_clade5 (1 AD)
9	2	* S. sonnei *	3.6.2	01-Jan	0/1	1 x Morocco	No
10	2	* S. flexneri *	3a	2/0	2/0	1 x Spain	UK_MSM sublineageA (15 AD); Australia_flexneri_BAPS3 (20 AD)
11	2	* S. sonnei *	3.7.18	2/0	01-Jan	1 x Peru	No
12	2	* S. sonnei *	3.6.1.1.2	2/0	2/0	None	BEL_I (11 AD)
13	2	* S. sonnei *	3.6.4	01-Jan	0/1	2 x Sri Lanka	No
14	2‡	* S. sonnei *	3.7.29.1.4.1	1/0^c^	1/0	None	BEL_II (4 AD); Spain_sonnei_clade1 (5 AD)

*Among male cases.

†No data about sex, MSM, travel available for three cases

‡No data about sex, MSM, travel available for one case.

SF, *Shigella flexneri *; SS, *Shigella sonnei *.

The largest cluster (cluster 1) within the study contained 15 isolates of *S. flexneri* serotype 1b, of which 10 isolates were from MSM, but had no link to 1 of the international reference isolates (Table 3). Three out of the remaining five *S. flexneri* clusters were related within 10–15 AD to internationally described reference isolates (clusters 5, 7 and 10), and cluster 8 was related within the cluster cut-off to a MSM-associated reference strain from Spain (Table 3).

Of the eight *S. sonnei* clusters, five (clusters 2, 3, 9, 11 and 13) were not related to international MSM reference genomes, although one of these clusters (cluster 3) consisted solely of MSM-associated isolates. Cluster 3 is of subclade 3.6.1.1, described as a successful clone with a triple mutation for quinolone resistance that has expanded globally [26, 28]. The other four were of subclades 3.7.18 and of subclades 3.6.2 and 3.6.4 that arose in the 1990s and were the first to acquire a chromosomal point mutation coding for ciprofloxacin resistance [26, 28]. The three remaining clusters (clusters 6, 12 and 14) were within 4–11 AD of internationally described MSM-related references (Table 3). Additionally, these clusters were of subclades that were described to be MSM-related previously [26].

### Antimicrobial resistance

In Table 4, the detected antimicrobial resistance markers are depicted, distributed over MSM-associated cases and non-MSM-associated cases (Table 4). Amongst isolates from non-MSM-associated cases, no resistance to cotrimoxazole was predicted for *S. flexneri* and five *S *. *sonnei* isolates (14 %) possessed both a *sul1* and one of the *dfrA* genes, coding for resistance against cotrimoxazole.

**Table 4.** Presence of antimicrobial resistance markers of the sequenced *Shigella* isolates by species (*n*=109), stratified by men who have sex with men (MSM) and females and males who do not have sex with men

	No or unkown MSM association				MSM-associated	
	* S. sonnei* (*n*=36)	* S. flexneri *	* S. boydii *	*S. dysent.*	* S. sonnei *	* S. flexneri *
		(*n*=24)	(*n*=1)	(*n*=1)	(*n*=19)	(*n*=28)
Quinolone	12 (33 %)	8 (33 %)	0	0	17 (89 %)*	5 (18 %)
*gyrA p.S83L*†	12	2	–	–	17	2
*gyrA p.D87G*†	8	1	–	–	15	0
*parC p.S80I*†	9	2	–	–	15	1
*qnrS1*‡	0	6	–	–	0	4
≥1 PM/gene	12	8	–	–	17	5
≥3 PM/gene	8	1	–	–	15	0
Aminoglycoside	35 (97 %)	19 (79 %)	1 (100 %)	1 (100 %)	19 (100 %)	28 (100 %)*
*aadA1*	11	13	1	1	4	27
*aadA5*	5	1	0	0	10	3
*aph(6)-Id (strB)*	28	8	1	0	16	2
aph(3’)-Ib (strA)	34	9	1	0	17	3
Macrolide	6 (17 %)	1 (4 %)	0	0	11 (58 %)*	12 (43 %)*
*erm(B)*	6	1	–	–	10	9
*mph(A)*	6	1	–	–	11	11
Sulphonamide	33 (92 %)	9 (38 %)	1 (100 %)	0	19 (100 %)	7 (25 %)
*sul1*	5	0	0	–	10	6
*sul2*	33	9	1	–	18	4
Trimethoprim	35 (97 %)	15 (63 %)	1 (100 %)	0	19 (100 %)	18 (64 %)
*dfrA1*	34	10	1	–	19	13
*dfrA5*	0	0	0	–	0	2
*dfrA7*	0	1	0	–	0	0
*dfrA14*	1	4	0	–	1	1
*dfrA17*	5	0	0	–	10	5
Tetracyline	25 (69 %)	19 (79 %)	1 (100 %)	1 (100 %)	16 (84 %)	27 (96 %)
*tet(A)*	25	6	1	0	16	0
*tet(B)*	0	14	0	1	1	27
Beta-lactam	7 (19 %)	18 (75 %)	0	1 (100 %)	10 (53 %)*	28 (100 %)
ESBL	1 (3 %)	13 (54 %)	0	1 (100 %)	3 (16 %)	28 (100 %)*
*bla*_*TEM-1B*_	6	6	–	0	8	4
*bla*_*OXA-1*_	0	13	–	1	0	27
*bla*_*CTX-M-27*_	1	0	–	0	3	1
Phenicol	0	13 (54 %)	0	1 (100 %)	0	26 (93 %)*
*catA1*	–	12	–	1	0	26
*floR*	–	1	–	0	0	0

*Significantly higher AMR in MSM-associated isolates (*P* ≤0.005).

†Chromosomal.

‡Plasmidal.

PM, point mutation.

Among MSM-associated isolates cotrimoxazole resistance was detected in all *S. sonnei* isolates (53 %) and 7 *S *. *flexneri* isolates (25 %) compared to non-MSM-associated isolates. This shows a statistically significant difference for resistance against co-trimoxazole for MSM-associated isolates compared to non-MSM isolates (*P*=0.002 for *S. sonnei *; *P*=0.018 for *S. flexneri *). Additionally, *S. sonnei* isolates from MSM-associated cases harboured significantly more resistance markers to ciprofloxacin (*P*<0.001) and macrolides such as azithromycin (*P*=0.002). MSM-associated *S. flexneri* possessed significantly more resistance markers against macrolide than non-MSM-associated *S. flexneri* (*P*<0.001, Table 4).

A combination of predicted resistance against cotrimoxazole, ciprofloxacin and azithromycin was not found within *S. flexneri* isolates. However, resistance against all recommended antimicrobials combined was found in 5 (14 %) non-MSM-associated *S. sonnei* isolates, but in 11 (58 %) of MSM-associated isolates, which is significantly higher (*P*=0.001).

MSM-associated *S. sonnei* clusters 3, 6 and 12 all contained three point mutations coding for ciprofloxacin resistance, and are assigned to subclade 3.6.1.1, known for the presence of these point mutations (Fig. 1). Clusters 6 and 12 have the same level 5 subclade, 3.6.1.1.2, described as MSM-associated [26]. A total of 13 out of 16 isolates from this study in subclade 3.6.1.1.2 seemed to be in possession of the MSM-associated pKSR100 plasmid and integron because of their AMR gene profile, with *ermB*, *mphA*, *bla_TEM-1_*, *dfrA17*, *sul1* and *aadA5*. The other MSM-associated cluster, 14, was assigned to subclade 3.7.29.1.4.1, also described earlier as being MSM-associated [26]. Both isolates in this cluster contained the ESBL gene *bla*_*CTX-M-27*_ and one point mutation coding for ciprofloxacin resistance, next to the *aadA1*, *strA*, *strB*, *sul1*, *sul2*, *dfrA1* and *tetA* genes (Fig. 1). One of the isolates also contained both azithromycin resistance markers *ermB* and *mphA*. None of the isolates in the non-MSM-associated *S. sonnei* clusters 2, 9, 11 and 13 contained point mutations or genes that coded for ciprofloxacin resistance, beta-lactamase and macrolides (Fig. 1).

In total, 24 out of 25 isolates in the *S. flexneri* clusters that contained one or more MSM-associated isolates (clusters 1, 5, 7, 8 and 10) harboured at least the genes *bla_OXA-1_*, *catA1*, *aadA1* and *tetB* (Fig. 2). This combination of genes is present on the *Shigella* resistance locus multidrug resistance element (SRL-MDRE), present in the ancestral strain of multiple MSM clades [5]. One of these isolates also seemed to have acquired the pKSR100 plasmid (*ermB*, *mphA*, *bla_TEM1_*) and integron (*dfrA17*, *sul1*, *aadA5*), both strongly associated with MSM clades of *S. flexneri* [5]. Cluster 4 is a travel-associated cluster, in which three out of four isolates possessed the gene combination *dfrA14*, *sul2*, *strA* and *strB*, described to be present in the pCERC-1 plasmid (Fig. 2) [5].

## Discussion

This pilot sentinel surveillance study showed that, within the Amsterdam region, more than half of the isolates from shigellosis patients are related to at least one other isolate based on cgMLST. A total of 14 clusters within the study period from February 2019 until October 2021 were identified, with cluster size varying from 2 to 15 patients. Ten of these clusters contained at least one MSM-associated isolate and eight clusters had at least one travel-associated isolate.

Almost half of the clusters were related to international MSM-associated reference genomes or belonged to an MSM-associated subclade earlier described for *S. sonnei* [26]. Additionally, we confirmed that isolates from different countries are related to each other, indicating the widespread presence of MSM-associated clades and their transmission within the MSM population in the Netherlands [5, 10–12, 14].

This study confirmed that the application of a genotyping scheme as proposed by Hawkey *et al.* for *S. sonnei* is vital in communication when putting clusters and outbreaks in a global context [26]. It was found that MSM-associated clusters that were considered to be new clusters in various countries belonged to the same subclades, indicating more widespread transmission [26]. In Belgium, several MSM-associated clusters, namely BEL-I, BEL-II, BEL-III and BEL-IV, were described as belonging to one of these MSM-associated subclades [11]. A study from Spain described that *S. sonnei* isolates from the Spanish clades 2, 3 and 4 were related to MSM-associated reference isolates from Australia and the UK, but genotyping was not performed [12]. In our study, however, we believe that the Spanish *S. sonnei* clade 1 belongs to subclade 3.7.29.1.4.1. This subclade was described as being internationally MSM-associated, with clade BEL-II also belonging to it, as well as cluster 14 identified in our study, indicating transmission of this subclade in at least Belgium, Spain and the Netherlands [11, 26]. One can hypothesize that it is already a well-established clade in the European continent, and if more genomic surveillance with application of the genotyping structure is performed, more isolates of this subclade will be detected. For *S. flexneri *, seven robust phylogenetic groups at lineage level were assigned in a former study, but a detailed genotyping scheme is not available [29]. A genotyping scheme for *S. sonnei* would be very useful to also put *S. flexneri* in a global context, because communication using serotypes has a low resolution and serotype switching has been described [30].

Although mild shigellosis does not need treatment, vulnerable patients and severe infections might require treatment, or treatment might be applied to shorten shedding periods [31, 32]. In Dutch treatment guidelines, treatment with cotrimoxazole or ciprofloxacin, or if necessary azithromycin, is recommended [31]. In the Netherlands, as described in other studies, markers for antimicrobial resistance are significantly more present in MSM-associated isolates, including mobile resistance elements such as SRL-MDRE and pKSR100 [5, 9, 14, 33], and both azithromycin resistance markers, *ermB* and *mphA* [33]. Additionally, in cluster 2 both MSM-associated isolates and isolates from women were found, indicating spread of MSM-associated strains to the broader population. This is especially relevant because of the higher prevalence of antimicrobial resistance that has emerged among MSM [5, 13, 14], which could potentially emerge into the broader population. However, drawing firm conclusions based on our study is challenging due to a limited study period and number of included isolates, making it difficult to determine the extent of spread. We anticipate that continuation of pathogen surveillance for *Shigella* will provide a better understanding of the extent of the spread of MSM-associated isolates into the broader population. This is especially relevant due to the potential public health impact of further spread of multidrug-resistant isolates [5, 13].

A limitation of this study was that part of the it was conducted during the COVID-19 pandemic, which caused a steep decrease in the number of shigellosis notifications because of various non-pharmaceutical control measures [34]. The decrease was most notable among travel-associated *S. sonnei* infections, which was most likely the result of travel restrictions. However, the number of shigellosis cases among MSM decreased less than that amongst women or non-MSM male cases. Possibly, this resulted in an atypical number or atypical composition of clusters compared to what would been have observed if there were no COVID-19 control measures in place. Additionally, the pandemic affected the power of this study, because even with a prolonged study duration, fewer samples were included than expected. In addition to causing a decreased number of shigellosis infections, the COVID-19 pandemic hindered the MHS in Amsterdam in performing interventions with respect to clusters because of the different focuses of the infectious disease department. Therefore, it is not clear if they would intervene more often than they would normally without the information from WGS data.

In conclusion, this pilot sentinel surveillance study met most criteria to implement a national genomic surveillance for *Shigella* isolates. It gave insights into the clustering of shigellosis cases in the Netherlands and within an international context. It confirms the worryingly widespread international transmission of (multi)drug-resistant strains, especially among the MSM population. This has already been described by studies in other countries, but is also present in a country with relatively low resistance rates for bacteria in general, such as the Netherlands [35]. This study indicates potential spreading of MSM-associated strains to females and males that do not have sex with men, although generalization is hampered by the relatively low sample size. Nevertheless, these study results warrant further monitoring of circulating *Shigella* strains in the Netherlands, especially those that are multidrug-resistant, as well as the spread of these strains from MSM to the broader population. Following the results from this pilot study, a national genomic surveillance programme for *Shigella* was established in the Netherlands in April 2022.

## Supplementary Data

Supplementary material 1
